# Modulatory Role of TPPP3 in Microtubule Organization and Its Impact on Alpha-Synuclein Pathology

**DOI:** 10.3390/cells11193025

**Published:** 2022-09-27

**Authors:** Judit Oláh, Attila Lehotzky, Tibor Szénási, Tímea Berki, Judit Ovádi

**Affiliations:** 1 Institute of Enzymology, Research Centre for Natural Sciences, 1117 Budapest, Hungary; 2 Department of Immunology and Biotechnology, Medical School, University of Pécs, 7624 Pécs, Hungary

**Keywords:** brain, TPPP proteins, microtubule, alpha-synuclein, pathophysiology, Parkinsonism

## Abstract

Parkinson’s disease is characterized by locomotion deficits, dopaminergic neuronal loss and alpha-synuclein (SYN) aggregates; the Tubulin Polymerization Promoting Protein (TPPP/p25 or TPPP1) is also implicated in these processes. The moonlighting and chameleon TPPP1 modulates the dynamics/stability of the multifunctional microtubule network by promoting its acetylation and bundling. Previously, we identified the microtubule-associated TPPP3, a homologue of TPPP1 lacking its N-terminus; however, its involvement in physiological or pathological processes was not elucidated. In this work, we have shown the modulatory role of TPPP3, similarly to TPPP1, in microtubule organization, as well as its homo- and hetero-associations with TPPP1. TPPP3, in contrast to TPPP1, virtually does not bind to SYN; consequently, it does not promote SYN aggregation. Its anti-aggregative potency is achieved by counteracting the formation of the TPPP1–SYN pathological complex/aggregation leading to Parkinsonism. The interactions of TPPP3 have been determined and quantified in vitro with recombinant human proteins, cell extracts and in living human cells using different methods including bifunctional fluorescence complementation. The tight association of TPPP3 with TPPP1, but not with SYN, may ensure a unique mechanism for its inhibitory effect. TPPP3 or its selected fragments may become a leading agent for developing anti-Parkinson agents.

## 1. Introduction

Parkinson’s disease (PD) is characterized by locomotion deficits, dopaminergic neuronal loss, mitochondrial dysfunctions and aggregates mainly comprised of alpha-synuclein (SYN) [1,2]. Multiple system atrophy (MSA) also belongs to synucleinopathies; it is characterized by neuronal loss and gliosis due to the formation of oligodendroglial cytoplasmic inclusions [3]. In these pathophysiological processes, in addition to SYN enrichment, the Tubulin Polymerization Promoting Protein (TPPP/p25 or TPPP1) is implicated as well [4]. We have reported the co-enrichment and co-localization of the two hallmark proteins, SYN and TPPP1, as visualized in neurons and oligodendrocytes (OLGs) in the brain samples of patients with PD and MSA, respectively, in spite of the fact that in the normal brain SYN and TPPP/p25 are expressed in distinct cell types ([5] and references therein). SYN is involved in a number of neurological processes, but its precise role displayed in neurons has not been evaluated in detail. Nevertheless, its role in neurotransmitter release and synaptic plasticity due to its association with synaptic vesicles has been suggested [6,7]. TPPP1 has been identified as a key player in the differentiation of the dividing progenitor OLG cells; it influences the microtubule dynamics and nucleation as well as the myelination in the CNS [8,9]. Accordingly, aberrant microtubule branching and shorter myelination in primary OLGs of TPPP1 knockout mice leads to axonal transport dysfunction and a degeneration manifested in motor coordination deficits [9].

We identified two homologues of TPPP1 lacking its N-terminus, denoted TPPP2 and TPPP3 (or TPPP/p18 and TPPP/p20) [10]. However, there is meagre information about their roles in either physiological or pathological events, except that TPPP3 is a tubulin-binding protein while TPPP2 is not. However, the active participation of TPPP1 in neurodegenerative processes due to its association with SYN is well-documented in the cases of synucleinopathies, in addition to its anti-mitotic activity [4,5,11]. Surprisingly, recent publications have reported the enrichment of TPPP3 in certain cancers [12,13,14,15,16,17,18,19,20,21,22,23,24,25,26]. The expression levels/functions of TPPP3 in tumor/cancer cells are tangled; it is largely dependent on the types of the tumors. TPPP1 expression counteracts the cell proliferation/division and it displays anti-cancer activity [5], while in the case of TPPP3, the protein seems to predominantly promote cell proliferation, migration and invasion, although there are conflicting data concerning even the same types of cancer (Table S1).

As summarized in Table S1, a number of data suggest higher TPPP3 expression, in contrast to TPPP1, in tumor cells of various lung, colorectal, ovarian tumors and clear cell sarcoma than that of normal ones [12,13,14,15,16,17,18,19]. Moreover, the down-regulation of TPPP3 at transcriptional levels inhibits tumor cell proliferation, migration and lung cancer growth in vivo [14]. Recently, higher RNA and/or protein TPPP3 levels have been found in glioblastoma, breast cancer, melanoma stem cells and endometrial cancer as well [20,21,22,23] where the higher TPPP3 expression levels are associated with poorer outcome and worse overall survival. The fact that the TPPP3 levels are distinct among the various cancers according to different databases/publications (Table S1) highlights the importance of carrying out in vitro and in vivo experiments along with such studies.

Independently of the cancer-related potency of TPPP3, the protein is involved in the developmental processes of the musculoskeletal system [27]. Recently, Zhang and co-workers have shown that TPPP3 is required for central and peripheral nerve regeneration [28]. The role of TPPP3 in promoting axon regeneration has also been suggested [29]. In addition, the participation of TPPP3 in palmitic acid-induced injury has been recently demonstrated in human endothelial cells [30]. The palmitic acid-induced oxidative damage comes off through the voltage-dependent anion channel 1 (VDAC1); the VDAC1 is a functional binding partner of TPPP3. The protein promotes the stability and activity of VDAC1, which is connected with the pathogenesis of cardiovascular disorders and metabolic-associated vascular complications [30]. Consequently, TPPP3-induced cell dysfunction may offer a potential therapeutic target in the treatment of vascular diseases [30]. An additional example of the functions of TPPP3 occurs in the course of implantation and pregnancy discovered in a mouse model [31,32]. TPPP3 is expressed in the endometrium, and its deletion leads to the implantation failure connected with the suppression of β-catenin as well. TPPP3 was identified as an interacting partner of β-catenin, the expression of which is regulated by TPPP3. Thus, TPPP3 plays a role in the embryo implantation and in the early pregnancy by modulating the β-catenin level, which is required for the embryo adhesion or the attachment process. The diverse functions of the TPPP3 protein published so far make it difficult to rank this protein into the therapy for cancerous diseases. 

The characterization of TPPP1 at molecular and cellular levels has been performed, and ligand and protein binding domains within the middle, flexible CORE region have been identified, which modulate the various independent (moonlighting) functions and high conformational plasticity (chameleon characteristics) of the protein [5,33]. For example, GTP and Zn^2+^ promote the dimerization of TPPP1 contributing to the stabilization of the microtubule network, while the binding of SYN or beta-amyloid to TPPP1 exerts its pathological functions. The unfolded N-terminus includes phosphorylation sites, while the C-terminus plays a crucial role in its association with tubulin/microtubules. These modifications display decisive roles in the physiological and pathological functions of TPPP1 (relevant reviews have been recently published) ([5] and references therein). 

In this work, we present experimental data on the various physiological and pathological functions of TPPP3 as compared to TPPP1, which allows for the evaluation of the similarities and differences of these two homologous proteins. We revealed that while both TPPP3 and TPPP1 tightly associate to tubulin/microtubules and modulate the organization of the microtubule network, they are distinct concerning their SYN binding and aggregative potencies. These studies may provide a better understanding of the TPPP-related pathological processes and may help to establish drug targeting for the treatment of distinct human diseases.

## 2. Materials and Methods

### 2.1. Sequence Comparison

Multiple alignments of sequences were conducted by the Clustal Omega program (https://www.ebi.ac.uk/Tools/msa/clustalo/) (European Molecular Biology Laboratory, European Bioinformatics Institute, EMBL-EBI, Hinxton, Cambridge, UK) (accessed on 25 July 2022) [34,35].

### 2.2. Prediction of Unstructured Regions

Sequences of human TPPP1 (UniProtKB O94811) and TPPP3 (UniProtKB Q9BW30) were submitted to the PONDR server (http://www.pondr.com) (accessed on 25 April 2022) using three different algorithms, namely VLXT, VL3 and VSL2 [36,37]. Access to PONDR was provided by Molecular Kinetics (IUETC, Indianapolis, IN, USA; e-mail: main@molecularkinetics.com) under the license from the WSU Research Foundation.

### 2.3. Prediction of Protein Structure

Structures of human TPPP1 and TPPP3 were predicted by AlphaFold (https://alphafold.ebi.ac.uk/) (European Molecular Biology Laboratory, European Bioinformatics Institute, EMBL-EBI, Hinxton, Cambridge, UK) (accessed on 25 July 2022) [38,39].

### 2.4. Antibodies

The used antibodies are listed and specified in Table 1. 

### 2.5. Production of Mouse TPPP3 (Clone No.8/B3-B7) Monoclonal Antibody

Female Balb/c mice (6 weeks old, Charles River) were immunized into each hind footpad and intraperitoneally (i.p.) with 30 μg of TPPP3 peptide–thyreoglobulin conjugate dissolved in complete Freund adjuvant, followed with two i.p. boosts in incomplete Freund adjuvant 21 and 14 days later. The splenocytes of the best responder animal were fused with Sp-2/0-Ag14 mouse myeloma cells (originally obtained from Flow Laboratories UK and maintained in our cell depository) according to the method described by Köhler and Milstein [41]. Supernatants from wells were assayed for the presence of TPPP3 antibodies by an indirect simple binding enzyme-linked immunosorbent assay (ELISA). The positive wells were cloned and further characterized.

### 2.6. Antibody Capture Indirect ELISA

TPPP3–bovine serum albumin (BSA) conjugate was used to determine the peptide binding ability of the clones. ELISA plates (NUNC Immuno plate: Thermo Fisher Scientific, Waltham, MA, USA) were coated with 50 μL of 1 μg/mL peptide conjugate or control proteins (e.g., BSA, gelatin) in coating buffer (50 mM sodium bicarbonate buffer pH 8.6) and incubated overnight at 4 °C. The nonspecific binding was blocked with 0.5% gelatin in phosphate-buffered saline (PBS) for 30 min. After three washing steps, the hybridoma supernatants or different dilutions of the purified monoclonal antibody were added, and the plates were incubated for 1 h at 37 °C, followed by adding horseradish peroxidase (HRP) labeled rabbit anti-mouse IgG. After incubation for 1 h at 37 °C, the plates were washed and developed with o-phenylenediamine (Merck). The reaction was stopped with sulfuric acid, and the absorbance at 490 nm was determined using an iEMS Reader MF (Thermo Fisher Scientific, Waltham, MA, USA). The isotype of the antibody was determined using a murine Ig isotyping ELISA kit (Merck), and it appeared to be IgG1.

### 2.7. Plasmids

The pcDNA3.1(-)-TPPP3, enhanced green fluorescent protein (EGFP)-TPPP3, pBiFC-V^N^-TPPP3 and pBiFC-V^C^-TPPP3 corresponding to the full-length human TPPP3 were amplified by polymerase chain reaction (PCR) using forward primer 5′-AGTTCTCGAGGCATGGCAGCGAGCACAGACATGG-3′ and reverse primer 5′- TTATGGATCCACTTCTTCACCTTGGCA-3′ and pDsRed2p20 [10] as a template. After digestion with XhoI and BamHI restriction enzymes, inserts were ligated into pcDNA3(-), pEGFPC1 and pBiFCs vectors (Novagen). The pBiFC-V^N^-TPPP1 and pBiFC-V^C^-SYN were as previously described [33,42]. Prokaryotic expression vector containing the insert for human TPPP1 and TPPP3 were prepared and purified as previously described [10,43]. Structures and sequences of all constructs were verified by restriction mapping and DNA sequencing.

### 2.8. Expression and Purification of Proteins

The hexaHis fusion human recombinant TPPP1 and TPPP3 proteins were expressed in E. coli BL21 (DE3) and isolated on a HIS-Select™ Cartridge (Merck P6611) as previously described [10,44]. The human recombinant SYN was prepared as previously described [45]. Tubulin was isolated from bovine brain [46].

The concentrations of the human recombinant proteins and tubulin were established by their absorbance at 280 nm with the following extinction coefficients (http://web.expasy.org/protparam) (Expasy server operated by the SIB Swiss Institute of Bioinformatics, Lausanne, Switzerland) (accessed on 25 April 2022): 10,095 M^−1^ cm^−1^ for TPPP1, 12,950 M^−1^ cm^−1^ for TPPP3, 5960 M^−1^ cm^−1^ for SYN and 50,310 M^−1^ cm^−1^ for tubulin. 

### 2.9. Brain Sample

Human adult brain tissue was obtained from the Human Brain Tissue Bank (HBTB, Semmelweis University, Budapest, Hungary) (https://semmelweis.hu/hbtb/) (accessed on 25 April 2022), which is a member of the BrainNet Europe II: #283, cerebellar cortex, female, age 85. Post-mortem interval was 8 h. HBTB’s activity is authorized by the Committee of Science and Research Ethic of the Ministry of Health, Hungary (ETT TUKEB: 189/KO/02.6008/2002/ETT) and the Semmelweis University Regional Committee of Science and Research Ethic (32/1992/TUKEB), and the Code of Ethics of the World Medical Association (Declaration of Helsinki).

### 2.10. Preparation of Cytosolic Brain Extract

Cytosolic extract from the cerebellar cortex of the human brain sample was prepared from human brain tissue. The tissue was homogenized in PBS buffer containing protease inhibitors (1 μg/mL leupeptin, 1 μg/mL pepstatin, 1 mM benzamidine and 10 μM 4-(2-aminoethyl)benzenesulfonyl fluoride hydrochloride) at a 1:3 ratio of tissue and buffer by glass/teflon Potter homogenizer (7 strokes). The homogenate was centrifuged at 17,000× *g*, 15 min, 4 °C, and then again at 100,000× *g*, 30 min, 4 °C. The obtained supernatant was used as cytosolic brain extract and subjected to Western blot. The protein concentration of the extract was measured by the Bradford method [47].

### 2.11. ELISA Experiments

The plate was coated with 5 μg/mL protein, as indicated in figure legends, overnight in PBS, and the wells were blocked with 1 mg/mL BSA in PBS for 1 h at room temperature. Then, the interacting partner was added at different concentrations, and its binding to the immobilized protein was quantified by a specific antibody followed by addition of the corresponding secondary antibody conjugated to HRP (Table 1). For the quantification of the immunopositivity, o-phenylenediamine with hydrogen peroxide as a substrate was applied, and the absorbance was read at 490 nm with an EnSpire Multimode Reader (Perkin Elmer, Waltham, MA, USA). The background value (no added proteins) was always subtracted. The apparent binding constant (Kd) was evaluated from the saturation curves by non-linear curve fitting (assuming single binding site hyperbola model) using Origin 2018 64-bit software (Northampton, MA, USA).

In the case of testing the specificity of the monoclonal TPPP3 antibody, the plate was coated with 5 μg/mL TPPP1 or TPPP3, and the monoclonal antibody was added at a serial dilution. 

In the case of the competitive ELISA, SYN (200 or 500 nM) pre-incubated for 30 min without or with TPPP proteins (500 nM) was added to the immobilized TPPP1. The bound SYN was quantified by a specific SYN antibody.

### 2.12. Turbidimetry Measurements and Pelleting Experiments

The assembly of tubulin (6 μM) was visualized by turbidity measurements in polymerization buffer (50 mM 2-(N-morpholino)ethanesulfonic acid buffer, pH 6.6, containing 100 mM KCl, 1 mM dithioerythritol, 1 mM MgCl_2_ and 1 mM ethylene glycol tetraacetic acid) at 37 °C with or without TPPP1 (3 μM) or TPPP3 (3, 9 or 12 μM). The polymerization was induced by the addition of TPPP proteins. The optical density was followed at 350 nm by a Cary 100 spectrophotometer (Varian, Walnut Creek, Australia). In the pelleting experiments, the polymerized samples (100 μL aliquot) were centrifuged (17,000× *g*, 15 min, 37 °C), and the pellet (P) and supernatant (S) fractions were separated. When indicated, the proteins alone were also prepared and centrifuged under the same conditions. The pellet fractions were resuspended in 100 μL polymerization buffer. The fractions were analyzed by sodium dodecyl sulfate polyacrylamide gel electrophoresis (SDS-PAGE). MM indicates the molecular weight marker (PageRuler Prestained Protein Ladder 26616, Thermo Scientific, Waltham, MA, USA).

### 2.13. Affinity Chromatography

SYN was immobilized to CNBr-activated Sepharose 4B (Amersham) according to the manufacturer’s instructions. The SYN bound to Sepharose was packed into columns. The binding capacity of a column was ~2 mg SYN per 1 mL Sepharose. The affinity column was equilibrated with phosphate buffer (10 mM phosphate buffer pH 7.4 containing 10 mM NaCl). TPPP1 or TPPP3 (0.25 mg) was loaded to the column, and then the column was washed with phosphate buffer. The bound proteins were eluted with phosphate buffer including 100 mM NaCl. After each experiment, the column was regenerated using 3 cycles of 0.1 M Na-acetate pH 4.0 buffer containing 0.5 M NaCl and 0.1 M tris(hydroxymethyl)aminomethane (Tris) pH 8.0 buffer containing 0.5 M NaCl. The eluted proteins were analyzed by 13.5% SDS-PAGE, stained with Coomassie Brilliant Blue R-250 containing 2-mercaptoethanol.

### 2.14. Cell Culture, Transfection and Manipulation

HeLa cells (ATCC^®^ CCL-2™) were grown in Dulbecco’s Modified Eagle’s Medium (high glucose) supplemented with 10% (*v*/*v*) fetal bovine serum (FBS), 100 μg/mL kanamycin and antibiotic antimycotic solution (all from Merck, Darmstadt, Germany; complete medium) in a humidified incubator at 37 °C with 5% CO_2_.

For cellular experiments, 5 × 10^4^ HeLa cells were plated in 24-well plates. For the co-localization studies, HeLa cells were transfected with EGFP-TPPP1 or EGFP-TPPP3 constructs using the Turbofect (Thermo Fisher Scientific, Waltham, MA, USA) transfection reagent according to the manufacture’s protocol. Then, the proteins TPPP1, TPPP3 and acetyl-tubulin were visualized by fluorescence microscopy using specific antibodies (Table 1).

The homo- and hetero-associations of the proteins were visualized by bimolecular fluorescence complementation (BiFC) technology. The coverslip-plated HeLa cells were transfected with the half-mVenus BiFC constructs of TPPP1, TPPP3 and SYN (100–100 ng for each V^N^-V^C^ pair) using Turbofect (Invitrogen) transfection reagent according to the manufacture’s protocol. As control, empty mVenus V^N^- and V^C^-plasmids were used. A 100 ng competitor unlabeled TPPP3 plasmid was co-transfected with mVenus BiFC constructs where mentioned. Nuclei were counterstained with Hoescht 33342 (Merck, Darmstadt, Germany). To visualize the microtubule structures and the BiFC signal by microscopy, the samples were fixed by cold methanol. The samples were then processed for tubulin immunocytochemistry and the fluorescent constructs were detected directly. 

### 2.15. Preparation of HeLa Cell Extract

Control cells or cells transfected with pEGFP-TPPP1 or pEGFP-TPPP3 (1 × 10^6^ HeLa cells plated in 6-well plates, incubated overnight) were washed by PBS, then suspended in situ in 50 mM Tris buffer pH 7.5 containing 100 mM NaCl and 0.5% NP-40 as well as protease inhibitors (1 μg/mL leupeptin, 1 μg/mL pepstatin, 1 mM benzamidine and 10 μM 4-(2-aminoethyl)benzenesulfonyl fluoride hydrochloride). Then, the samples were centrifuged at 17,000× *g*, 10 min, 4 °C. The obtained supernatant was used as a cellular extract and subjected to ELISA or Western blot. The protein concentration was determined by the Bradford method [47].

### 2.16. ELISA on Cells (cELISA) for Detection of the Transfected EGFP-TPPP1 and EGFP-TPPP3

HeLa cells were inverse transfected in suspension by the referred plasmids according to the manufacturer’s protocol, and then sampled from the transfected cell suspension on a 96-well tissue culture plate (2 × 10^4^ cells/well). As a positive control of acetylation, Trichostatin A (TSA) was added into parallel, non-transfected samples for 1 h before the end of the experimental period. Non-transfected cells were used as cellular background samples. For comparison, we used EGFP tag as an identical marker of transcription for both plasmids as control and reference value by cELISA in parallel samples.

After overall 24 h of experimental period, the plate was fixed by ice-cold methanol for 10 min. Then, the wells were rehydrated by PBS, and blocked with PBS containing 0.1% Triton-X-100 and 1% FBS for overnight at 4 °C. Then, the plate was incubated with monoclonal acetylated tubulin antibody or GFP antibody, and then with the corresponding IgG-peroxidase conjugate (Table 1). Both antibodies were in PBS buffer containing 1% FBS and 0.1% Triton-X-100 and incubated for 1 h at room temperature. Between each incubation step, the wells were washed thrice with PBS for 5 min. The presence of acetylated α-tubulin (Lys40) or EGFP was detected using o-phenylenediamine as described in the ELISA method above.

### 2.17. Western Blot

The proteins were separated by 13.5% SDS-PAGE, then were electro transferred onto Immobilon-PSQ transfer membranes and subjected to Western blot to quantify the protein of interest. After blocking, the protein bands were visualized and quantified by using specific antibodies (Table 1). Antibody binding was visualized by the appropriate IgG–peroxidase conjugate by a Bio-Rad ChemiDoc MP Imaging system and its ImageLab 4.1 software (Bio-Rad, Hercules, CA, USA) using Immobilon Western substrate (Merck, Darmstadt, Germany). For densitometric analysis, the intensity of the bands was analyzed using the measure command and subtracting background values by ImageJ 1.49.

Amido black staining (0.1% *w*/*v* amido black, 25% *v*/*v* isopropanol and 10% *v*/*v* acetic acid solution) was also carried out for the protein bands on the membrane.

### 2.18. Immunofluorescence Microscopy

Confocal images were acquired with a Zeiss LSM 710 microscope (Zeiss, Jena, Germany) controlled by the LSM Zen 2010 B SP1 software using oiled 40 × NA = 1.4 Plan Apo objectives and the following lasers: Diode laser at 405 nm for Hoescht 33342, Argon laser at 488 nm for EGFP, Argon laser at 514 nm for BiFC signal and HeNe laser at 543 nm for Alexa 546. Detection range of fluorophores was determined by the software using built-in emission parameters considering the fluorophores; signals were acquired one by one.

Tiff formats of the images were created from the original pictures (2048 × 2048 pixels, 72 dpi, 8-bit lsm images). Complex images were created with Adobe Photoshop 2022 CC (San José, CA, USA).

The BiFC intensity per cell was counted for each sample by an observer blinded to the experimental conditions determined by the National Institutes of Health ImageJ software. The whole territory of minimum 50 cells was outlined by the Freehand Line tool and analyzed by taking the sum of the grey values of the pixels in the selection (integrated density) and by subtracting the background.

### 2.19. Statistical Analysis

All values are presented as the mean ± SD (standard deviation) of at least 3 independent experiments. Statistical comparisons were performed with one-way ANOVA followed by Tukey’s test for multiple comparisons or Student’s *t*-test using Origin 2018 64-bit. Values were considered to be significant if the calculated *p* value was < 0.05 (* *p* < 0.05, ** *p* < 0.01, *** *p* < 0.001 and **** *p* < 0.0001).

## 3. Results and Discussion

### 3.1. Characteristics of the Human TPPP3 Protein

**Figure 1:**
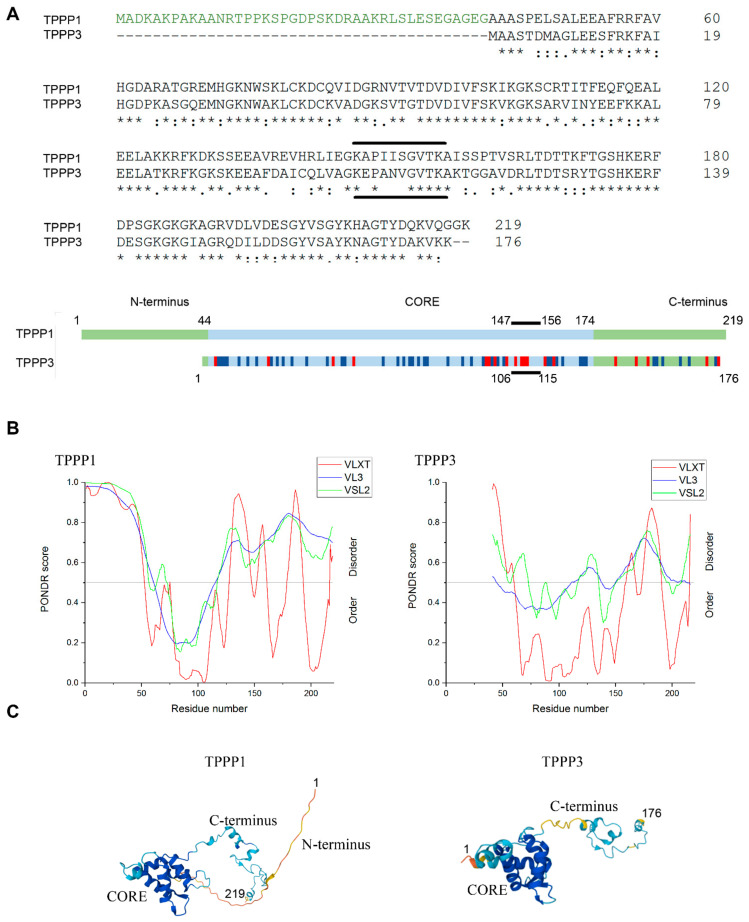
Primary and secondary structures of TPPP1 and TPPP3. (**A**) Multiple alignment of the human TPPP1 and TPPP3 by Clustal Omega (https://www.ebi.ac.uk/Tools/msa/clustalo): identical (*) and similar (: or .) residues. Similar and different residues in the scheme are indicated by dark blue and red, respectively. The TPPP1 segment involved in SYN binding is indicated by lines. (**B**) PONDR prediction of the terminal disorder segments of the proteins evaluated by three algorithms (VLXT, VL3 and VSL2) (http://www.pondr.com). Disorder prediction values (PONDR scores) of a given residue are plotted against the residue number. The residue numbers of TPPP1 are shown for the sake of comparison. Residues considered to be disordered above threshold of 0.5. (**C**) Predicted structures by AlphaFold (https://alphafold.ebi.ac.uk). AlphaFold produces a per-residue confidence score (pLDDT) between 0 and 100 (pLDDT > 90 very high, dark blue; 90 > pLDDT > 70 confident, cyan; 70 > pLDDT > 50 low, yellow).

The human TPPP1 (219 amino acids) was the first identified member of a new eukaryotic protein superfamily [10,48]. The TPPP family is present in mammals including human and other vertebrates. The human TPPP3 (176 amino acids) shows a high degree of homology with TPPP1 disregarding the N-terminus of TPPP1 [10]. The comparison of the primary sequences of the two TPPP proteins, as illustrated in Figure 1A, highlights the lack of the first 42 amino acids, as well as the definitive dissimilarity in the middle (CORE) region of TPPPs, previously identified as the SYN binding motive of TPPP1 (Figure 1A).

The 3D structure of TPPP3, but not that of the disordered TPPP1, was determined by multinuclear NMR [49]; nevertheless, the disorder prediction for both proteins was performed using a neural network-based predictor PONDR. Using three different algorithms [36,37], the analysis revealed that the middle CORE region of TPPP1 and its homologue appear to be ordered, while the C-terminal segments of both proteins are largely disordered (Figure 1B). Searching the Protein Data Bank (https://www.rcsb.org/) (accessed on 25 July 2022), the structure of human TPPP3 is available (PDB DOI: 10.2210/pdb2JRF/pdb), but there is no data for human TPPP1. Therefore, we used AlphaFold, a state-of-the-art application for the prediction of 3D protein structures [38,39], for the comparison of the structure of the two homologues. This program confidently predicts secondary structures such as alpha-helices, which are localized in the middle region of both proteins, while their C-terminal segments are unfolded (Figure 1C), highlighting the similarity of these two proteins.

It should be added that the lack of the N-terminal segment may not cause significant difference in the binding to the tubulin/microtubules since the tubulin-binding domain has been localized within the C-terminus of TPPP1 [5]. However, the impact of TPPP3 on SYN pathology could be distinct from that of TPPP1 considering the alterations in the sequences in the SYN binding segment (147–156 amino acids).

### 3.2. Quantification of the TPPP Levels in Human Brain

In the past, the TPPP1 level was determined and quantified in the bovine brain followed by its isolation [48]. It was also reported that TPPP1-positive cells are abundant in the white matter, but the protein is also expressed in the cerebral cortex and the fiber-like structures in the CA3 region of hippocampus [50]. Evidence has been provided for the specific expression of TPPP1 in OLGs in the case of a normal human brain, which is crucial for their differentiation [8]. According to the Human Protein Atlas (https://www.proteinatlas.org) (accessed on 25 July 2022), TPPP3 can be found in the cerebral cortex, hippocampus and hypothalamus, and it is localized to ependymal cells and a subset of glial cells (https://www.proteinatlas.org/ENSG00000159713-TPPP3/brain) (accessed on 25 July 2022). 

To quantify the endogenous TPPP3 level in the human brain, a TPPP3-specific antibody is needed; specific polyclonal and monoclonal TPPP1 antibodies had been produced earlier and used successfully [4,40]. We produced a monoclonal TPPP3 antibody against human recombinant TPPP3 injected into mice as described in the Material and Methods Section. The specificity of the antibody was tested by both ELISA and Western blot (Figure S1) as carried out in the case of TPPP1 [40]. As illustrated in Figure S1, the monoclonal TPPP3 antibody displayed high immunoreactivity against TPPP3 in both tests, and cross-reactivity was not detected in either case. These data revealed the high specificities of the antibodies, indicating that they are appropriate for the quantification of TPPP levels in the human brain. 

**Figure 2:**
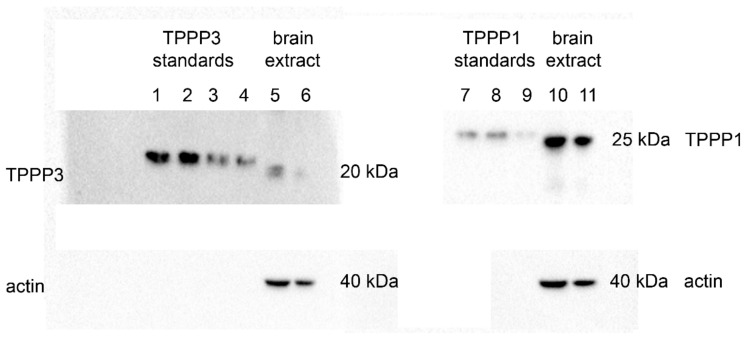
TPPP3 and TPPP1 levels in human brain as determined by Western blot using monoclonal TPPP3 antibody and polyclonal TPPP1 antibody. Both recombinant TPPP1 and TPPP3 containing His-tag (lanes 1–4 and 7–9) and brain extracts (22 and 11 μg total protein of the extracts on lanes 5/10 and 6/11, respectively) were loaded to the gel and subjected to Western blot. TPPP3 standards: 40, 32, 24 and 16 ng (lanes 1–4), TPPP1 standards: 16, 12 and 8 ng (lanes 7–9).

Human brain tissues (from the cortical region) were obtained from the Human Brain Tissue Bank founded by Prof. Palkovits, and the TPPP proteins were determined as described in the Materials and Methods Section; TPPP3 and TPPP1 were detected and quantified by Western blot. Figure 2 shows the immunopositivities of both tested proteins; either the recombinant (control) or the endogenous TPPP protein. Figure 2 shows the number of the loaded proteins used for the quantification of the two proteins resulting in 4.33 ng TPPP1/μg total protein versus 0.57 ng TPPP3/μg total protein. This finding reveals the simultaneous occurrence of the TPPP proteins as well as provides evidence for their different (one order of magnitude) intracellular concentrations in the tested samples.

### 3.3. TPPP3, like TPPP1, Is a Microtubule-Associated Protein 

Previously the interaction of the human recombinant TPPP3 with tubulin/microtubules was established by circular dichroism, differential scanning calorimetry, surface plasmon resonance and visualized by transmission electron microscopy [10]. These studies have revealed that both TPPPs are microtubule-associated proteins (MAPs), and their interactions cause conformational alterations as well as induce the formation of intact-like and aberrant tubulin/microtubule assemblies [10,51,52]. 

**Figure 3:**
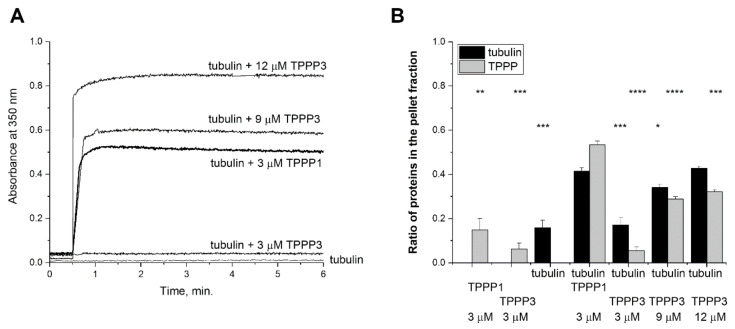
Interaction of TPPP3 and TPPP1 with tubulin. (**A**) TPPPs-induced tubulin polymerization was measured by turbidimetry; 3 μM TPPP1 or 3, 9 or 12 μM TPPP3 was added to 6 μM tubulin. A representative experiment is shown. (**B**) The extent of the polymerization was quantified by pelleting experiments following the centrifugation of the samples (17,000 g, 10 min, 37 °C); the pellet (P) and supernatant (S) fractions were loaded to 13.5% SDS-PAGE and the partition of the proteins in the pellet (P) fraction was quantified by densitometric analysis. Statistical comparisons were performed with two-sided, unpaired Student’s *t*-test, as compared to the control (tubulin + 3 μM TPPP1) (* *p* < 0.05, ** *p* < 0.01, *** *p* < 0.001 and **** *p* < 0.0001).

We aimed to compare the tubulin binding efficacy and tubulin polymerization/assembly potencies of TPPP3 and TPPP1. ELISA experiments were carried out with immobilized TPPP3 or TPPP1 on the plate, and tubulin was added at various concentrations; the bound tubulin was detected by a specific antibody (Figure S2A). It was found that both TPPP proteins bind to tubulin with high and comparable affinities. We also tested the functional consequences of their hetero-associations. The turbidity measurements show the tubulin polymerization potency of TPPP3 at different concentrations; accordingly, a three-fold higher TPPP3 concentration (9 μM) produces a time course similar to that of TPPP1 (Figure 3A). At the maximum values of the turbidity, caused by the added TPPP proteins, the partition of the tubulin in the supernatant (soluble tubulin) and the pellet (polymerized microtubules) phases were analyzed as described in Section 2. As shown in Figure 3B and Figure S2B, the tubulin alone predominantly appears in the supernatant, while the presence of TPPP1 or TPPP3 altered the partition of the tubulin. At an identical concentration (3 μM), more tubulin appears in the pellet phase in the presence of TPPP1 than in the case of TPPP3. Under these conditions, the pelleting of TPPP3 either in the absence or presence of tubulin is less pronounced than that of TPPP1, suggesting the decreased polymerization/assembly potency of TPPP3. This finding corresponds to the binding data [10], which showed a slightly higher binding affinity for TPPP1 than for TPPP3 determined by surface plasmon resonance (K_d_ = 1.0 ± 0.1 and 0.32 ± 0.03 μM for TPPP3 and TPPP1) and by circular dichroism (EC_50_ = 8.0 and 2.5 μM for TPPP3 and TPPP1), respectively.

### 3.4. Homo- and Hetero-Association of TPPP3

**Figure 4:**
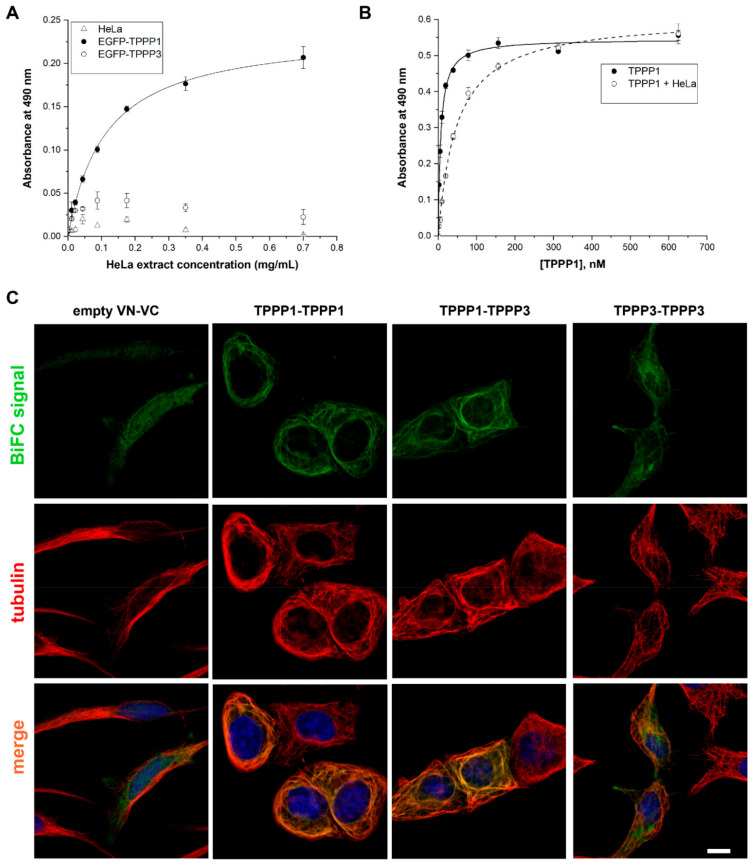
Homo- and hetero-association of TPPP3. (**A**) Bindings of TPPPs (EGFP-TPPP3 (○), EGFP-TPPP1 (●) and EGFP alone (∆)) expressed in HeLa cells to the immobilized TPPP3 were quantified by addition of specific GFP antibodies. (**B**) Effect of HeLa extract on the TPPP3–TPPP1 interaction. Human recombinant TPPP1 were added at different concentrations to the immobilized TPPP3 without (●) and with (○) HeLa extract (0.32 mg/mL), and the binding of TPPP1 was quantified by monoclonal TPPP1 antibody; Kd = 6.53 ± 0.38 nM for TPPP1 and 47.7 ± 2.4 nM for TPPP1 in the presence of HeLa. (**C**) Representative images of the intracellular homo- and hetero-dimerization of TPPP3 and TPPP1 in HeLa cells were visualized by BiFC technology as described in Section 2. The microtubule network was stained with Alexa546 (red). Nuclei are counterstained with Hoescht 33342 (blue). Scale bar: 5 μm.

TPPP1 occurs in dimeric/oligomeric forms, which play an important role in the microtubule bundling/stabilization [42,53,54]. The homo- and hetero-dimerization of TPPP3 were studied by different methods using human recombinant proteins, cell extracts expressing EGFP-TPPP1 and EGFP-TPPP3 and living human cells. In the ELISA experiments, TPPP3 was immobilized on the plate, and the extracts of HeLa cells transfected with pEGFP-TPPP3 or pEGFP-TPPP1 were added in different concentrations. The bound EGFP-labelled TPPPs were detected by specific EGFP antibodies (Table 1). As shown in Figure 4A, the intracellularly expressed TPPPs bind to the immobilized TPPP3; however, a large difference in their binding can be detected. In another set of experiments, the effect of the cell extract (not transfected HeLa cells) on the hetero-association of TPPP1 and TPPP3 was characterized. As shown in Figure 4B, TPPP1 binds to TPPP3 in the presence of the HeLa extract, although to a lesser extent. 

Next, direct evidence of the formation of the homo- and hetero-associations of TPPP3 in living human cells is substantiated by the application of BiFC technology that was successfully used for the demonstration of the homo- and hetero-association of TPPP1 [33,42]. The green BiFC signal indicates the interaction of two partner proteins fused separately to the N- and C-termini of the split Venus proteins by bringing the N- and C-domains of the fluorescent Venus in close proximity, so that they can be detected by fluorescent microscopy [42]. As shown in Figure 4C and Figure S3, TPPP3 forms both homodimers as well as heterodimers with TPPP1. In addition, the fluorescent microscopic data revealed the co-localization and the bundling effect of the TPPP dimers on the microtubule network, although this effect seems to be less pronounced in the case of the TPPP3 homodimer as compared to its heterodimer. Previously, we reported the crucial role of TPPP1 dimerization in the stabilization of the microtubule network, in which the unfolded terminals of TPPP1 play crucial roles [42]. In fact, the TPPP1-derived microtubule stabilization has a physiological impact on the development of projections in the course of the differentiation of the progenitor OLGs involved in the construction of the myelin sheath [8].

### 3.5. Effect of TPPP3 on the Acetylation Level of the Microtubule Network

**Figure 5:**
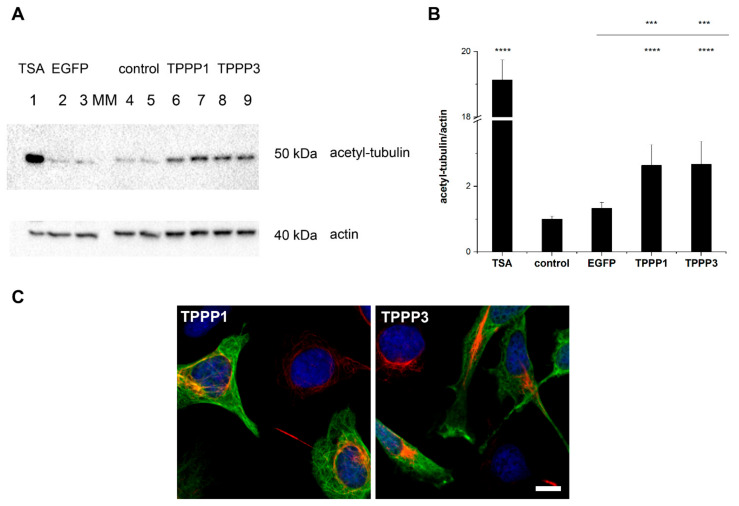
The promoting effects of TPPP3 and TPPP1 on the tubulin acetylation. (**A**) In Western blot experiment, the tubulin acetylation level was quantified by acetyl-tubulin antibody in HeLa cells transfected with pEGFP-TPPP3 or pEGFP-TPPP1 constructs. Control cells do not express TPPP proteins, only empty EGFP or TSA as a tubulin deacetylase inhibitor was added as well. (**B**) Quantification of the acetylation-promoting effects of TPPP proteins on the microtubule network of HeLa cells by Western blot. Data are presented as the mean ± SD. Statistical comparisons were performed with one-way ANOVA followed by Tukey’s test, as compared to the control or as the lines indicate (*** *p* < 0.001 and **** *p* < 0.0001). (**C**) Representative images illustrate the TPPP3- and TPPP1-derived acetylation of the microtubule network in HeLa cells as detected by fluorescence microscopy. The cells were transfected with pEGFP-TPPP3 (green) or pEGFP-TPPP1 (green); acetyl-tubulin was detected by specific acetyl-tubulin antibody (red). Nuclei are counterstained with Hoescht 33342 (blue). Scale bar: 5 μm.

Previously, we showed that TPPP1 regulates the dynamics and stability of the microtubule network through its acetylation-enhancing activity by inhibiting the cytosolic tubulin deacetylase enzymes, histone deacetylase 6 and sirtuin-2 [43,55]. We tested the effect of TPPP3, as compared to that of TPPP1, on the microtubule acetylation by Western blot in HeLa cells expressing TPPP3 and TPPP1, as described in the Materials and Methods Section. As shown in Figure 5A,B, there is no significant difference in the acetylation level of the TPPP1- and TPPP3-expressing cells.

**Figure 6:**
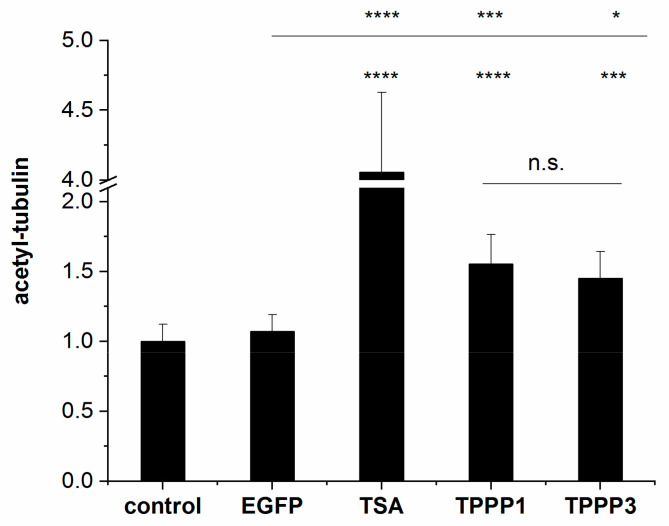
Quantification of the acetylation-promoting effects of TPPP proteins on the microtubule network in living human cells by cELISA assay. HeLa cells were transfected with pEGFP-TPPP3 or pEGFP-TPPP1 or treated with the tubulin deacetylase inhibitor TSA, as described in the Materials and Methods Section. The EGFP and the acetyl-tubulin levels were detected by specific antibodies. The values for medium (in absence of cells) were subtracted in all cases. The acetyl-tubulin levels are presented as the mean ± SD. Statistical comparisons were performed with one-way ANOVA followed by Tukey’s test, as compared to the control or as the lines indicate (* *p* < 0.05, *** *p* < 0.001 and **** *p* < 0.0001). n.s.: not significant.

We quantified the acetylation-enhancing potency of the two TPPPs by cELISA experiments with living HeLa cells expressing EGFP-TPPP3 or EGFP-TPPP1, and the acetyl-tubulin level was determined on the basis of the immunopositivity against the acetyl-tubulin antibody, as described in the Materials and Methods Section. The intracellular levels of the EGFP signal were also established for the normalization of the acetylation levels. In addition, the effect of TSA, a tubulin deacetylase inhibitor [56], was used as a positive control. Control cells were used as well. As shown in Figure 6, TSA significantly increased the tubulin acetylation level as expected. The quantitative data for the tubulin acetylation was obtained after normalization with respect to the expression levels of the EGFP-TPPP proteins. These data show that both TPPP1 and TPPP3 significantly increase tubulin acetylation; their effects are comparable.

### 3.6. Distinct Pathological Functions of the TPPPs Proteins

TPPP1 and SYN, hallmark proteins of synucleinopathies, are co-localized and co-enriched in cytoplasmic inclusions characteristic for PD and MSA [4,5]. TPPP3 is involved in different cancerous processes (cf. Table S1). Little information has been reported concerning the connection between the cancer and neurodegeneration, but there are some recent findings that PD patients present a reduced risk for certain cancers, but an increased risk for melanoma [57,58]. There is no information available yet for the possible role of TPPP3 in neurodegeneration. The association of human recombinant TPPP1 and SYN has been characterized by a ~100 nM binding constant; in their hetero-association, the 147–156 amino acid segment of TPPP1 is involved [33,44]. The alignment of the primary sequences of the two TPPP proteins shows differences in the SYN binding region of TPPP1 (cf. Figure 1). We investigated the binding of TPPP3 to SYN using different methodologies, such as affinity chromatography, ELISA and BiFC technology.

In the affinity chromatography experiments, TPPP3 or TPPP1 were added to SYN immobilized onto the column; the eluted fractions were tested for the partition of bound/unbound TPPPs by SDS-PAGE, as described in the Material and Methods Section. As shown in Figure S4, only a small amount of TPPP3 was retarded on the column, while TPPP1 was predominantly detected in the eluted fractions.

**Figure 7:**
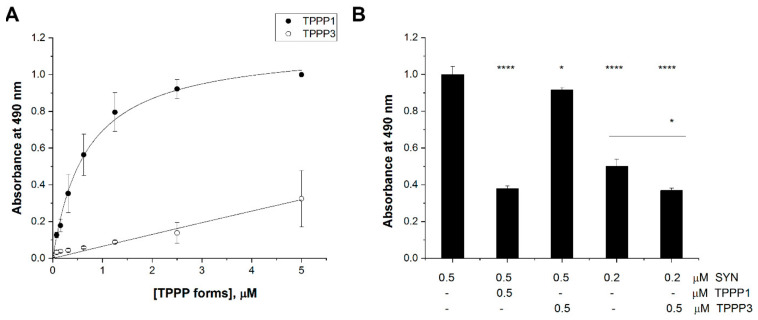
Interaction of TPPP3 with SYN compared to that of TPPP1. (**A**) In an ELISA experiment, the plate was coated with SYN, then human recombinant TPPP1 (●) or TPPP3 (○) was added at different concentrations and the bound proteins were detected by specific antibodies. The binding affinities were evaluated by curve fitting, assuming simple hyperbolic saturation: 673 ± 58 nM for TPPP1. (**B**) In the competitive ELISA experiments, SYN alone or with TPPP3 was added to the immobilized TPPP1 and the binding of SYN was quantified by SYN antibodies. The normalized results are presented as means ± of the standard deviation (SD). Statistical comparisons were performed with two-sided, unpaired Student’s *t*-test, as compared to the control or as the lines indicate (* *p* < 0.05 and **** *p* < 0.0001).

ELISA experiments were carried out to quantify these interactions. In one experimental setup, the plate was coated with SYN, then TPPP3 or TPPP1 was added at different concentrations, and the bindings of TPPPs to the immobilized SYN were detected by specific antibodies (Figure 7A). TPPP3 binding to the SYN was much weaker than that of TPPP1; in fact, no significant binding affinity of TPPP3 can be detected under the condition used (Kd = 673 ± 58 nM for TPPP1). In addition, a competitive ELISA experiment was also carried out, and the inhibitory potency of TPPP3 on the interaction of TPPP1 with SYN was analyzed (Figure 7B) following the addition of the premixed SYN and TPPP1 or TPPP3 at different concentrations to the immobilized TPPP1 (cf. Section 2). The inhibitory potency of TPPP3 on the formation of the pathological TPPP1–SYN complex is definitive; the mechanism responsible for the inhibition may be due to the assembly of the two TPPPs, because TPPP3 does not have a SYN-binding capacity.

**Figure 8:**
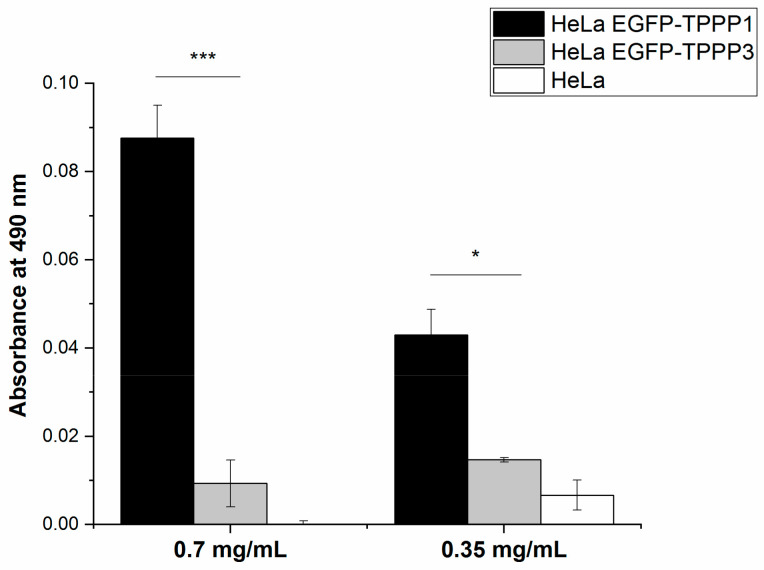
Interaction of SYN with TPPP3 as compared with that of TPPP1. In an ELISA experiment, extracts of HeLa cells transfected with pEGFP-TPPP3 (grey) and pEGFP-TPPP1 (black) were added to the immobilized SYN. HeLa extract (white) was used as control. The bound proteins were quantified by EGFP antibodies. Statistical comparisons were performed with two-sided, unpaired Student’s *t*-test, as compared as the lines indicate (* *p* < 0.05 and *** *p* < 0.001).

Next, ELISA experiments were performed with extracts of Hela cells transfected with pEGFP-TPPP1 or pEGFP-TPPP3, which was added to the immobilized SYN at different concentrations. Following incubation, the bound TPPPs were determined by specific EGFP antibodies. As illustrated in Figure 8, the binding of TPPP1 to SYN is much higher than that of TPPP3, which is negligible. Consequently, TPPP3 could not promote SYN assembly. These data reveal that TPPP3 does not display an aggregation-promoting potency.

### 3.7. Anti-Aggregative Potency of TPPP3

**Figure 9:**
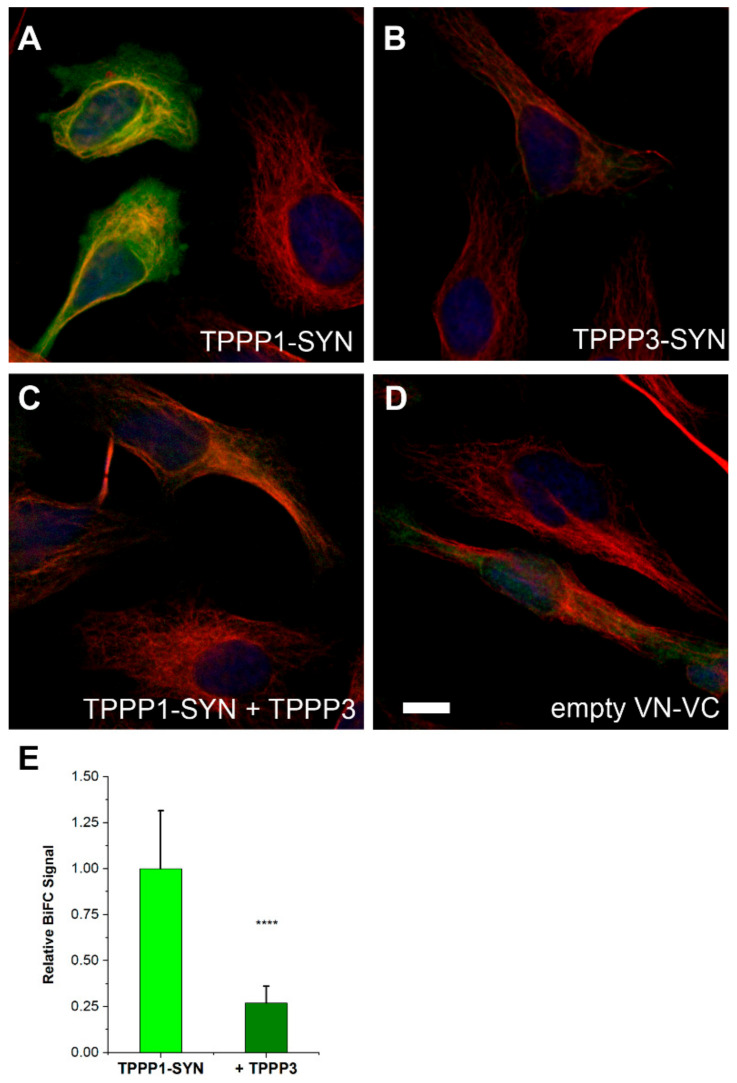
The effect of TPPP3 on the association of TPPP1 and SYN in living human cell model. (**A**,**B**) The associations of SYN and TPPP1, but not that of TPPP3, are visualized by BiFC technology. The cells were co-transfected with TPPP1 and SYN (**A**,**C**) or with TPPP3 and SYN (**B**) fused to mVenus, or with empty mVenus (**D**) BiFC constructs; the microtubule network was stained with Alexa546 (red). (**C**) In a competition experiment, cells were co-transfected with unlabeled TPPP3 simultaneously with the Venus constructs of TPPP1 and SYN. Nuclei are counterstained with Hoescht 33342 (blue). Scale bar: 5 μm. (**E**) Quantification of the BiFC signal by detecting the fluorescence intensity of the cells without and with TPPP3. Data were compared by two-sided, unpaired Student’s *t*-test, **** *p* < 0.0001.

In living human cell experiments, we exploited the lack of the direct interaction of TPPP3 with SYN, and its hindering effect on the formation of the pathological TPPP1–SYN interaction was investigated by employing BiFC technology. As illustrated in Figure 9 and Figure S5, the pathological association of SYN with TPPP1 (BiFC signal) (Figure 9A) is proceeded by the simultaneous transfection of mVenus^N^- and mVenus^C^- constructs of SYN and TPPP1. However, a significantly lower BiFC signal was observed for TPPP3 and SYN than for TPPP1 and SYN (Figure 9B and Figure S5). Control experiments revealed that the simultaneous expression of the empty mVenus^N^ and mVenus^C^ did not result in a significant BiFC signal (Figure 9D).

However, when TPPP3 was also transfected into the cells in addition to the Venus constructs of the TPPP1–SYN complex, it significantly hindered the formation of the pathological complex as demonstrated by the quantification of the fluorescence intensities (BiFC signals) of the individual cells (Figure 9C,E). These data correspond to those obtained in vitro by a competitive ELISA experiment (cf. Figure 7B). The negligible binding of TPPP3 to SYN and the firm association of TPPP3 with TPPP1 can provide an effective mechanism ensuring the destruction of the pathological complex.

## 4. Conclusions

The goal of this project was to establish the characteristics of TPPP3, which can account for the distinct behaviors of TPPP1 and TPPP3 (lacking the N-terminus) under physiological and pathological circumstances. The comparative studies have shown that the highly homologous TPPPs are tubulin/microtubule-binding proteins, which promote the tubulin polymerization and acetylation, resulting in the stabilization of the microtubule network. However, the aggregation-promoting activity is characteristic only of TPPP1, but not of TPPP3; the former is involved in the development of neurodegenerative disorders, namely synucleinopathies. This feature is seemingly unrelated to the anti-mitotic activity of TPPP1, but it correlates with our observation that TPPP1 does not occur in rapidly dividing cancerous (e.g., HeLa) cells. 

As introduced in the Introduction Section of this paper, in the cases of many cancerous cells, the TPPP3 level is extremely high, excluding its anti-mitotic potency. The low or no acetylation-promoting potency of TPPP3, in contrast to that of TPPP1 causing the stabilization of the microtubule network, may be a basic course of the distinct pathological features of the two TPPP proteins; however, their acetylation-promoting potencies were found to be comparable. Despite this issue, the cancerous cells with a high TPPP3 level can display uncontrolled (extensive) cell division with active contribution of the microtubule-based dynamic action of the spindles. 

**Figure 10:**
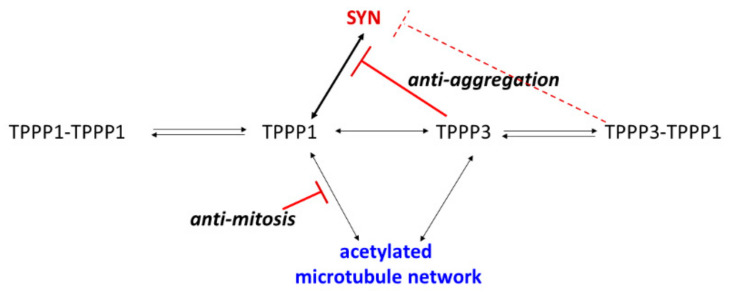
The physiological and pathological interactions of TPPP1 and TPPP3.

We have identified unexpected characteristics of the homologous TPPP3. While the two TPPP proteins display similar binding and functional activities as MAPs, the key difference in their properties is their affinities to the brain-specific SYN, the aggregation of which is extensively promoted by TPPP1, which can exclude the extensive division of the cells (Figure 10). This plausible notion seems to be supported by clinical observation, namely that PD patients have a reduced risk for cancer [57,58]. 

However, surprisingly, TPPP3 has a high affinity to TPPP1, which may have an important pathological impact. A major message of the present project is that the inhibitory effect of TPPP3 resulted in the destruction of the pathological TPPP1–SYN complex. Previously, we identified the interface of the TPPP1–SYN complex, marking potential drug target at its contact surface [33,44,59]. The destruction of the pathological complex by TPPP3 is not necessarily due to its targeting of the interface, but the firm associative ability of the TPPP proteins can contradict the TPPP1–SYN association. This mechanism results in that SYN may display its physiological function; in addition, the excess SYN may be degraded via the autophagy and by ubiquitin–proteasome systems, which cannot be achieved when SYN is in a complexed form [60]. Furthermore, no side effect of TPPP3 is expected, since TPPPs may not have a function in neurons. The next innovative goal of this project is the identification of the TPPP3 segment, which may act as a leading molecule for developing effective anti-Parkinson agents.

## Figures and Tables

**Table 1:** Antibodies used for the experiments.

Antibody	Company	Catalog Number	Dilution
Rat polyclonal anti-TPPP1	[4]		1:5000
Mouse monoclonal anti-TPPP1	[40]		1:1000
Mouse monoclonal anti-TPPP3	cf. 2.5 and 2.6		1:2000
Mouse monoclonal anti-alpha-tubulin, clone DM1A	Merck (Darmstadt, Germany)	T9026	1:5000
Mouse monoclonal anti-acetyl-tubulin, clone 6-11B-1	Merck (Darmstadt, Germany)	T6793	1:1000 cELISA; 1:5000 Western blot
Rabbit polyclonal anti-GFP	Thermo Fisher Scientific (Waltham, MA, USA)	A11122	1:2000
Mouse monoclonal anti-SYN	Merck (Darmstadt, Germany)	S5566	1:5000
Mouse monoclonal anti-beta-actin	Thermo Fisher Scientific (Waltham, MA, USA)	MA1-140	1:5000
Anti-rat IgG, HRP-linked	Merck (Darmstadt, Germany)	A9037	1:5000
Anti-mouse IgG, HRP-linked	Merck (Darmstadt, Germany)	A2554	1:2000 cELISA; 1:5000 other
Anti-rabbit IgG, HRP-linked	Thermo Fisher Scientific (Waltham, MA, USA)	32260	1:2000 cELISA
Anti-mouse IgG, Alexa-546 linked	Thermo Fisher Scientific (Waltham, MA, USA)	A11003	1:1000

## Data Availability

All primary data can be found in Data_for_figures.xlsx Excel file in Supplementary Materials.

## Supplementary Materials

The following supporting information can be downloaded at: https://www.mdpi.com/article/10.3390/cells11193025/s1, Figure S1: Testing the specificity of the monoclonal TPPP3 antibody by ELISA (A) and Western blot (B); Figure S2: Interaction of TPPP3 and TPPP1 with tubulin; Figure S3: Homo- and hetero-associations of TPPP3; Figure S4: SDS-PAGE analysis of TPPP proteins loaded to SYN affinity column; Figure S5: The effect of TPPP3 on the association of TPPP1 and SYN in living human cell model. Table S1: The role of TPPP3 in cancer.
