# Supplementary material for: Modulatory Role of TPPP3 in Microtubule Organization and Its Impact on Alpha-Synuclein Pathology
Source: Cells. 2022 Sep 27;11(19):3025. doi: 10.3390/cells11193025 (PMC9564178; doi:10.3390/cells11193025)
Supplement: Supplementary file 1 [file cells-11-03025-s001.zip › SupplementaryMaterial.pdf]

Supplementary Materials

MODULATORY ROLE OF TPPP3 IN MICROTUBULE ORGANIZATION AND ITS IMPACT ON ALPHA-SYNUCLEIN PATHOLOGY

A

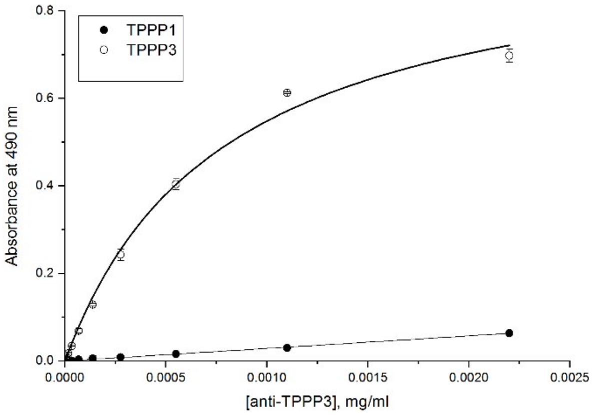

B

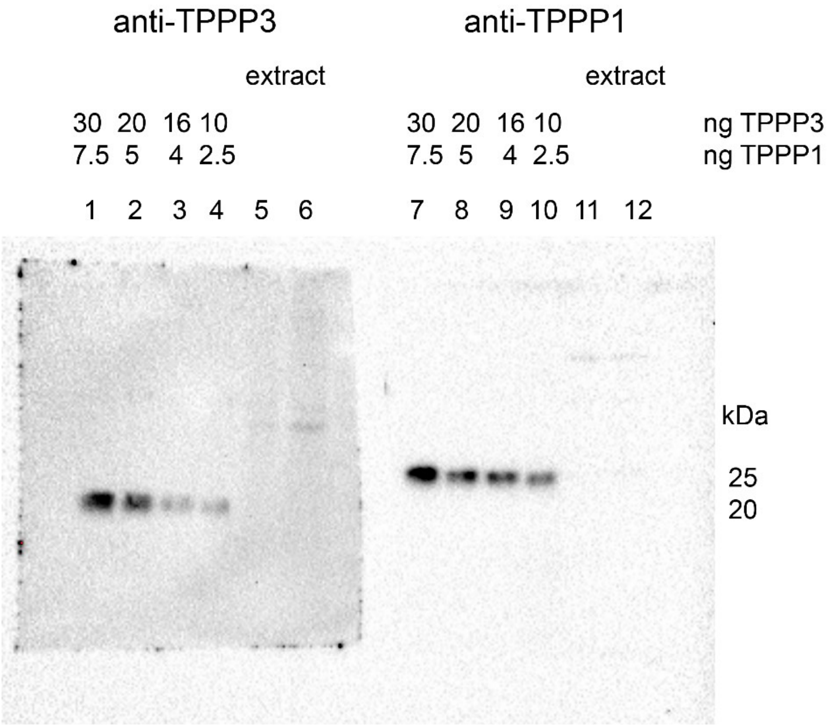

Figure S1. Testing the specificity of the monoclonal TPPP3 antibody by ELISA (A) and Western blot (B). (A) The plate was coated with human recombinant TPPP1 (●) or TPPP3 (○), then monoclonal TPPP3 antibody was added in a serial dilution. The apparent Kd value of the antibody is 0.784 ± 0.077 μg/ml for TPPP3. (B) Western blot experiment. Both recombinant TPPP1 and TPPP3 were loaded to lanes 1-4 and 7-10; HeLa extract was loaded to lanes 6 and 12 (20 μg total protein). The blots were developed by monoclonal TPPP3 and polyclonal TPPP1 [4] antibodies, respectively.

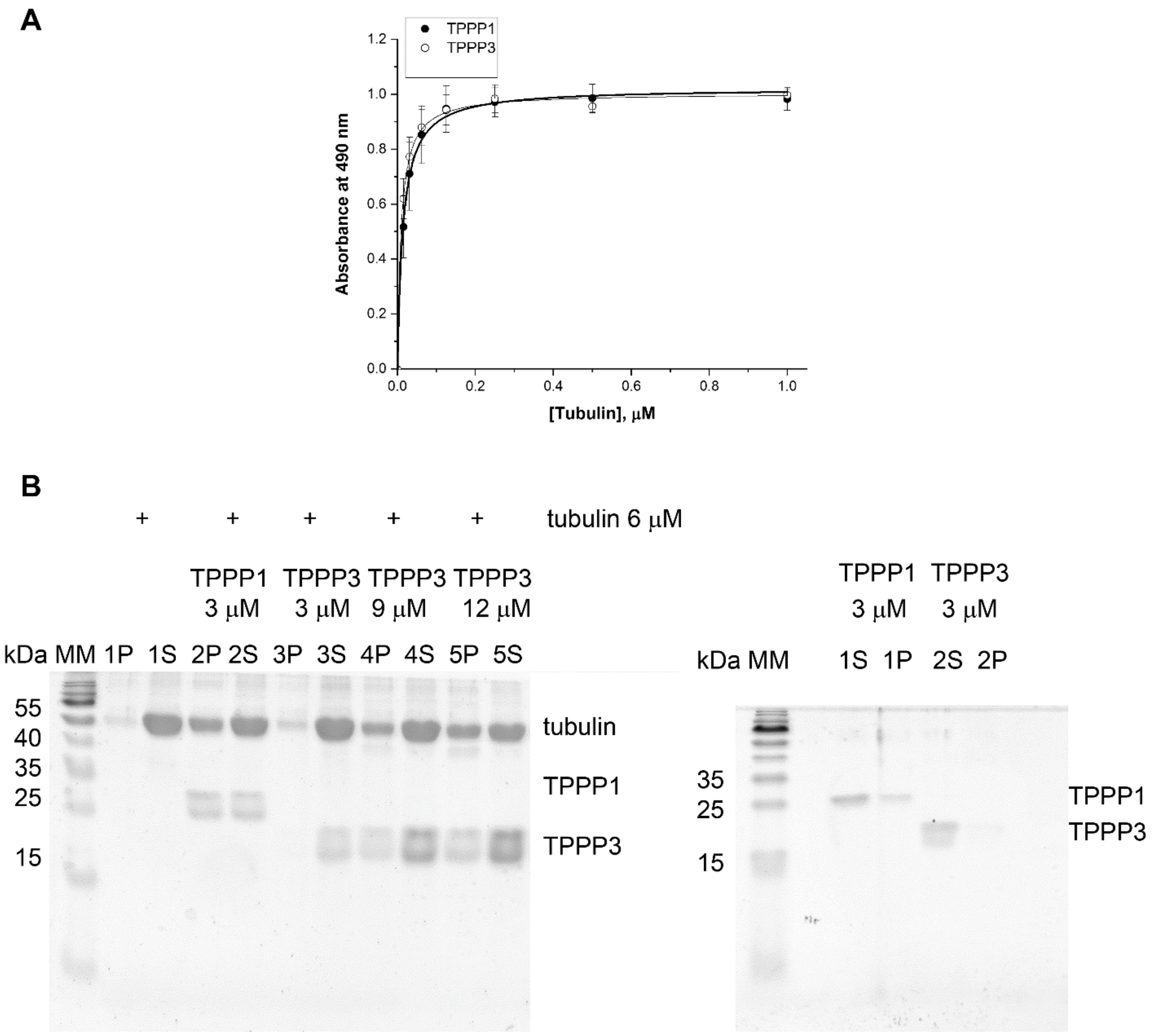

Figure S2. Interaction of TPPP3 and TPPP1 with tubulin. (A) In the ELISA experiment, the plate was coated with TPPP1 (●) or TPPP3 (○), then tubulin was added at different concentrations. The bound tubulin was detected by tubulin antibody as described in the Materials and methods. The results are presented as means ± the standard deviation (SD). The binding affinities were evaluated by curve fitting assuming simple hyperbolic saturation: 14.0 ± 1.0 nM for TPPP1 and 9.4 ± 0.6 nM for TPPP3. (B) In the pelleting experiment the samples obtained from the polymerization assays (Fig. 3A) were centrifuged (17000 g, 10 min, 37°C), and the pellet (P) and supernatant (S) fractions were loaded to 13.5% SDS-PAGE and analyzed.

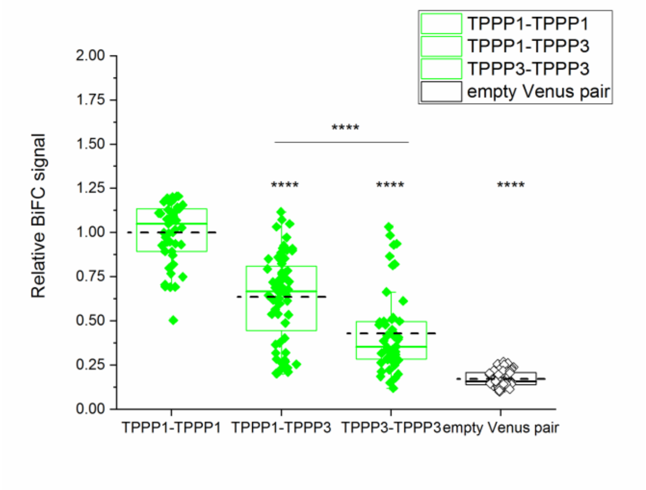

Figure S3. Homo- and hetero-association of TPPP3. Quantification of the BiFC signal by detecting the fluorescence intensity of the cells. Box extends from the 25th to 75th percentile with the middle solid green and the dashed black lines representing the median and the mean, respectively. Data were compared by two-sided, unpaired Student's t-test, **** $p < 0.0001$.

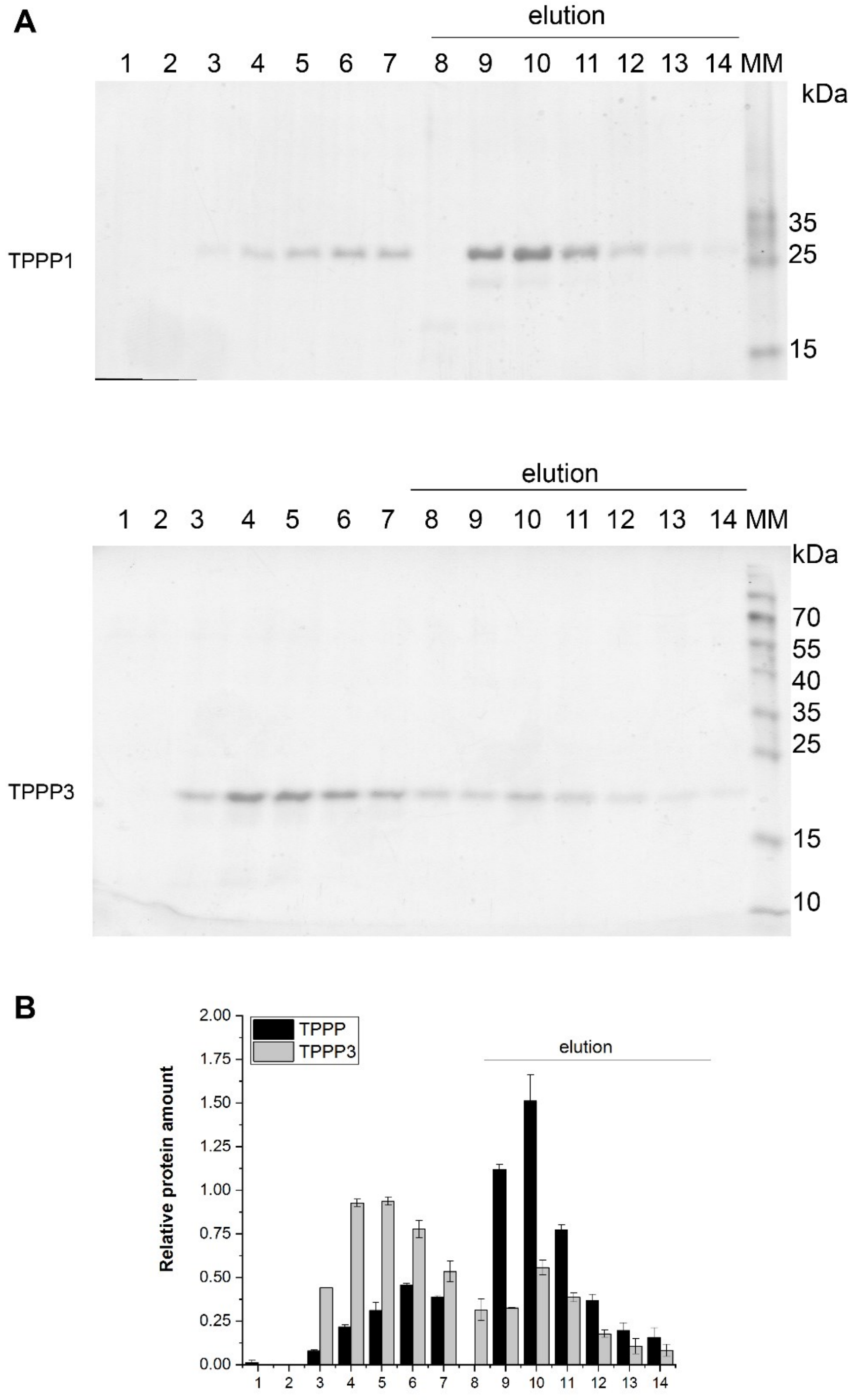

Figure S4. SDS-PAGE analysis of TPPP proteins loaded to SYN affinity column. (A) TPPP1 or TPPP3 was loaded to the column as described in the Materials and methods, the unbound and bound (eluted) fractions are shown. Representative images. (B) The relative amount of proteins in the fractions as determined by concentration measurement.

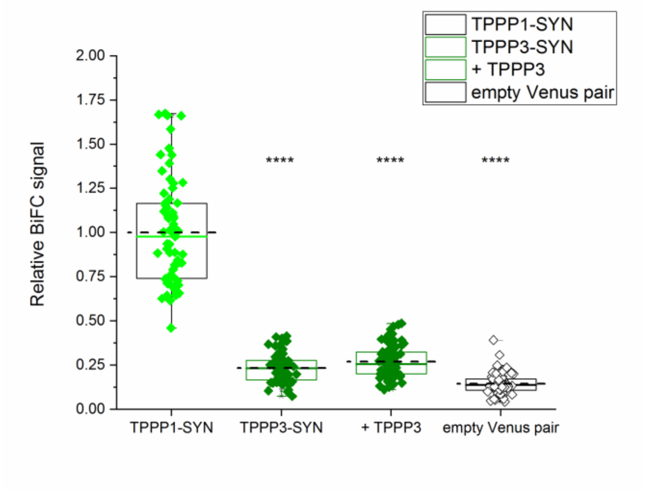

Figure S5. The effect of TPPP3 on the association of TPPP1 and SYN in living human cell model. Quantification of the BiFC signal by detecting the fluorescence intensity of the cells. Box extends from the 25th to 75th percentile with the middle solid green and the dashed black lines representing the median and the mean, respectively. Data were compared by two-sided, unpaired Student's t-test, **** $p < 0.0001$.

Table S1. The role of TPPP3 in cancer.

Cancer	TPPP3	Level	Functional consequences	Reference
cancerous HeLa cells	RNA		RNAi depletion: suppressed proliferation with cell cycle arrest	[12]
Lung (Lewis carcinoma)	RNA		RNAi depletion: inhibition of tumor growth	[13]
non-small cell Lung carcinoma (NSCLC)	Protein	high	shRNA knockdown: cell proliferation inhibition; TPPP3 depletion: lung cancer growth inhibition in vivo	[14]
non-small cell Lung carcinoma (NSCLC)	RNA, Protein	high	TPPP3 promotes cell proliferation, invasion and tumor metastasis	[15]
Lung	Protein	high		[16]
Colorectal	RNA, Protein	high	knockdown: inhibition of cell proliferation, migration and invasion; high TPPP3: lower overall survival rate	[17]
Ovarian	RNA	high	10x overexpression	[18]
Clear cell sarcoma	RNA, Protein	high		[19]
Glioblastoma	RNA		high TPPP3 expression: worse overall survival	[20]
Breast cancer	Protein	high	Silence of TPPP3: suppressed cell proliferation, invasion and migration; high TPPP3 expression: poor outcomes	[21]
Melanoma stem cells	RNA, Protein	high		[22]
Endometrial cancer	RNA, Protein	high	TPPP3 knockdown: suppression in cell proliferation, migration and invasion	[23]
Pancreatic ductal adenocarcinoma	Protein		higher TPPP3 level: indication of long survival	[24]
Nasopharyngeal carcinoma	RNA, Protein	low	TPPP3 overexpression: inhibition of cell proliferation and invasion	[25]
Head and neck squamous carcinoma	RNA, Protein	low	low TPPP3 expression: associated with poor prognosis; TPPP3 is associated with immune infiltration levels	[26]
